# Editorial: New insights into prostate cancer: new biomarkers, molecular mechanisms, and therapeutic approaches

**DOI:** 10.3389/fendo.2024.1453065

**Published:** 2024-07-24

**Authors:** Anna Perri, Vittoria Rago, Guadalupe Maya-Núñez

**Affiliations:** ^1^ Department of Experimental and Clinical Medicine, Magna Graecia University, Catanzaro, Italy; ^2^ Department of Pharmacy, Health and Nutritional Sciences, University of Calabria, Rende (CS), Italy; ^3^ Unidad de Investigación Médica en Medicina Reproductiva, Coordinación de Investigación en Salud, Instituto Mexicano del Seguro Social, Mexico City, Mexico

**Keywords:** prostate cancer (PCa), castration-resistant PCa (CRPC), biomarkers, androgen receptor, G protein-coupled estrogen receptor (GPER), positive surgical margins, bioactive compounds

Prostate cancer (PCa) is the second most frequent cancer among men worldwide (1). PCa is considered curable when it is localized, but when it metastasizes the clinical treatment is complex. Androgen deprivation therapy (ADT) is a regular treatment for PCa patients; however, some of them develop castration-resistant PCa (CRPC), and despite several new treatment options, metastatic CRPC has a poor prognosis with a survival rate below 2–3 years (2).

The present Research Topic aims to present new emerging evidence in diagnostics and treatment of PCa.

The Prostate specific antigen (PSA) is a poor indicator of early PCa, particularly if its levels are between 4 and 20 ng/ml. Among other PCa serum markers, Sialic acid (SA) represents a potential candidate for tumor aggressiveness, as demonstrated in other types of cancer (3). Sun and Yan reported that in patients with PSA values between 4 and 20 ng/mL, the serum biochemical index, including SA, is a potential prediction marker for PCa. The authors suggested that SA levels could prevent unnecessary biopsies and biopsy-related morbidities. Furthermore, Sun et al., demonstrated that the serum SA levels in patients without treatment are positively correlated with bone metastases, consequently proposing that elevated SA levels before surgery could be an indicator of increased malignancy risk and advanced cancer stages. Cathepsins play a crucial role in preserving cellular homeostasis; however, under inflammatory conditions, they can drive cancer progression and other diseases (4, 5). The Mendelian randomization study of Cao et al., provided new insights into the role of cathepsins in the diagnosis and treatment of benign prostate diseases (BPD), as they offered the first evidence of a genetic causal link between cathepsins and BPD.

The identification of new specific markers associated with cancer recurrence is critical in the management of PCa patients. The autophagy-related gene expression levels have great potential in predicting tumor recurrence risk and evaluating the response to treatment in PCa patients (6, 7). Kang et al., built a risk model using four anoikis-related genes that effectively predict the risk of recurrence and survival outcomes in PCa patients, confirming the clinical value of in-depth investigation of anoikis-related genes in PCa.

Drug resistance CRPC mechanisms due to AR splice variants, mutations, or glucocorticoid receptor (GR) substitution of AR function, is a common cause of treatment failure. The search for pharmacological agents to overcome this cancer resistance conducted to plant extracts research (8). Lam et al., demonstrated the efficacy of the bioactive compound YIV-818-A in overcoming drug resistance caused by AR variants and GR, by inhibiting AR and GR activities. Some studies elucidated the molecular mechanism underlying the anti-tumor effects of curcumin on both androgen-sensitive and -insensitive prostate cancer cells (9). The meta-analyses conducted by Wang et al. showed a favorable association between PCa and curcumin treatment, highlighting that the curcumin dosage potentially impacted the treatment efficacy. Overall these findings further reinforce the concept that dietary supplements could be used as chemoprevention agent for PCa, although further studies are needed to ascertain their use in the clinical practice.

G protein-coupled estrogen receptor (GPER) plays an important role in tumor development and metastasis, by activating different signaling pathways (10, 11). The GPER-mediated signaling in PCa is the hedgehog (Hh) pathway and has not been widely investigated. This signaling-pathway activation is associated with aggressiveness, metastasis, and relapse in triple-negative breast cancer, occurring *via* glioma-associated oncogene homolog (GLI) transcriptional factors (12). The study of Rico-Fuentes et al., performed on PCa samples with different prognostic grades demonstrated that GPER is highly expressed in the nucleus and its expression increases with the cancer progression. Furthermore, GPER’s expression correlates with pGLI3 nuclear expression across different stage groups, suggesting that GPER could represent a new marker of tumor aggressiveness.

Insulin growth factor (IGF) signaling plays a key role in the development and progression of PCa. Consequently, targeting IGF signaling has been considered a potential therapeutic strategy for this cancer (13). Elemam et al., provided an overview of the main evidence on prostate tumorigenesis regulation *via* the IGF system, highlighting that drugs targeting the IGF family could represent a promising therapeutic approach in the management of advanced PCa.

Many researchers found a complex and close association between the immune system and PCa (14). Still, few studies have investigated the association between immune cells and the risk of PCa. Hao et al., identified immune cell phenotypes significantly associated with PCa, contributing to open new insights for exploring potential immunotherapeutic targets in PCa.

An increasing number of articles highlight the positive surgical margin (PSM) as a relevant parameter to make decisions for adjuvant treatment and predict patient outcomes after radical prostatectomy (RP), mainly in patients with localized PCa (15). Therefore, the factors influencing PSM should be considered by urologists during operation, regardless of the surgery approach (i.e. robot-assisted, laparoscopic, and open RP). The monocentric study of Wang et al. showed that PSM risk was increased in patients receiving hormonal therapy before the surgery compared with patients without treatment, highlighting that the resection extent should be accurately evaluated. In the past, the RP was rarely the first option for advanced PCa, but thanks to the introduction of robot-assisted RP, it has been reported that advanced PCa patients respond well to ADT therapy and have better progression-free survival after cytoreductive prostatectomy, suggesting that surgery might be an effective treatment option also for advanced PCa (16). Li et al. built a clinical features-based prognosis model demonstrating the benefits of surgery in patients with advanced PCa, and emphasizing that its accuracy may offer some reference on clinical decision-making.

In conclusion, this Research Topic presents interesting new evidence in the field of precision medicine for PCa diagnosis and treatment. Although relevant progress has been made, further studies are needed to better understand how to overcome castration resistance, find new predictors of tumor recurrences, and improve the survival rate.

## Author contributions

AP: Writing – review & editing, Writing – original draft. VR: Writing – review & editing, Writing – original draft. GM-N: Writing – review & editing, Writing – original draft.
